# Editorial

**Published:** 2015-12-01

**Authors:** K. Vesal

**Affiliations:** 1 Life time member of the Irainian Academy of Medical Sciences, Tehran, Iran

Regarding the increasingly diverse topics in the field of biomedical physics and engineering, the editorial team of the JBPE had decided to publish “topic issue” from the beginning of 2014. 

Publishing topic issues involves several challenges such as publication delays. However, JBPE managed to solve this problem through Online First section. Accordingly, editor-in-chief and publishing office of the JBPE pursued a successful endeavor and published the Online First section to reduce the publication delays. 

A previously ignored point revealed itself after through and conclusive inspections of the results. Topic issues in e-journals are not as beneficial as they are in printed journals. In fact, we believe that there should be a new perspective on the philosophy of e-journals. E-journal readers do not find their desired papers based on the topic of the issue; they search, find and select articles solely based on the keywords and their own research interests. Therefore, the rest of the articles that are being published along with a specific article in a topic issue journal, will not benefit our readers. In other words, cohesion of the papers in a specific topic issue seems to be unnecessary.

Meanwhile, the importance of the Online First section of the journal has emphasized in the editorial of the JBPE and we will strengthen this section further in the subsequent issues. 

The second topic that had been pursued in the past three years at JBPE was the fast track. In the fast track, articles that are derived from graduate theses will be verified through their supervisor’s cover letter and enter a fast track review process which will take less than four weeks. The restriction in the number of the reviewers had prevented JBPE to officially declare this policy in the journal’s website in the past three years. However, by joining new editors to the editorial board of the JBPE and the registration of the journal in new indexings, the journal will officially implement its fast track policy from the beginning of 2016. 

Since the editorial management of the articles in fast track will be financially demanding, fast track process include partial payment of the review and the editorial expenses. 

Meanwhile, with the help of the JBPE’s generous sponsors, we will maintain our commitment to the open access and free of charge submission policies of the journal. Based on the editorial board’s approval and by considering different restrictions that exist for researchers and scientists of the developing countries, JBPE will keep its policy to waive all or most of the revision expenses.

Last but not least, regarding the international scope of the JBPE, our journal willingly encourages researchers in the field of biomedical physics and engineering to contact the editorial office for paper review, submission of solicited papers and membership in the editorial board. 

The world had experienced several sad events at the end of the 2015 but I wish a happy, peaceful and prosperous 2016 for all. 

